# Hypertrophic pulmonary osteoarthropathy with primary lung cancer

**DOI:** 10.3892/ol.2014.2022

**Published:** 2014-04-02

**Authors:** XINYU QIAN, JING QIN

**Affiliations:** 1 Department of Chemotherapy, Hangzhou First People’s Hospital, Hangzhou Cancer Hospital, Hangzhou, Zhejiang 310002, P.R. China; 2 Department of Chemotherapy, Zhejiang Cancer Hospital, Hangzhou, Zhejiang 310022, P.R. China

**Keywords:** lung cancer, hypertrophic pulmonary osteoarthropathy, bone scintigraphy, incidence

## Abstract

Hypertrophic pulmonary osteoarthropathy (HPO) is a rare paraneoplastic syndrome that is frequently associated with lung cancer; however, the incidence of clinically apparent HPO is not well known. The clinical data of 6,151 patients with advanced lung cancer between January 1996 and December 2008 were retrospectively analyzed in Zhejiang Cancer Hospital (Hangzhou, China). Data pertaining to the presentation of HPO, diagnosis, treatment, pathology, follow-up and clinical course were documented. HPO was confirmed using bone scintigraphy by the identification of symmetrical, abnormally high uptake in the joints, and/or long bones with digital clubbing in the extremities as well as joint pain. The clinical characteristics were investigated based on clinical and pathological records. The patients were presenting with lung cancer for the first time and had not received treatment. Among the 6,151 lung cancer patients, 115 (1.87%) demonstrated an abnormally high uptake on bone scintigraphy and digital clubbing in the extremities combined with joint pain. A total of 109 patients received surgery or chemotherapy treatment and 92 exhibited improved symptoms. The improvement rate of HPO was lower in the non-surgery compared with the surgery patients (77.4 vs. 91.1%; P=0.049). Bone scintigraphy was repeated in 85 patients within 3–6 months, and the HPO symptoms improved in 70 patients. The present retrospective study indicated that 1.87% of patients with lung cancer showed characteristics that were identified as HPO. The majority of the patient symptoms and the bone scintigram of HPO improved as a result of treatment.

## Introduction

Hypertrophic pulmonary osteoarthropathy (HPO), also known as the Pierre Marie-Bamberger syndrome, was first described by Bamberger in 1889 (1), and is distinguished by painful, swollen joints, digital clubbing and periostitis (2,3). HPO is an uncommon clinical syndrome that may be associated with various pulmonary and non-pulmonary diseases, including the hepatopulmonary syndrome of advanced liver or cirrhosis cyanotic heart diseases (4). HPO may be an indicator of malignant disease and various types of cancer also induce HPO, including primary malignancies or metastasis carcinomas (5–7). As a gold standard for detecting bone metastases of malignancies, bone scintigraphy is a highly sensitive method for the diagnosis of HPO (8). The typical scintigraphic presentation is a diffuse, symmetrically increased uptake in the diaphysis and metaphysis of tubular bones, with a distinctive double stripe or parallel track sign (7). According to previous studies, the incidence of HPO ranges from 0.7 to 17% (9–13). To the best of our knowledge, few studies have investigated the incidence of HPO in a large series of lung cancer patients.

The present study investigated the incidence of HPO and the clinical characteristics of patients with lung cancer and HPO based on a large population of lung cancer patients from China.

## Patients and methods

Between January 1996 and December 2008, 6,151 patients with lung cancer underwent bone scintigraphy at the Zhejiang Cancer Hospital (Hangzhou, China). A total of 115 consecutive, unselected patients with lung cancer were included in the present study. The study was approved by the ethics committee of the Zhejiang Cancer Hospital (Hangzhou, China). The clinical characteristics were investigated based on available clinical and pathological records. Lung cancer staging was performed for all patients according to the seventh tumor-node-metastasis classification (14). The patient selection criteria were as follows: i) Pathologically proven primary lung cancer. The patients who had other disorders (for example, multiple bone metastases, arthropathy with known causes, such as rheumatoid arthritis or heart disease, abnormal venous stasis in the lower extremities, or thyroid and pituitary dysfunction) that may have led to an incorrect diagnosis of HPO were excluded. HPO was diagnosed based on bone scintigraphy findings at the time of lung cancer diagnosis. ii) All the patients received bone scintigraphy; the bone scintigrams were performed with 740 MBq ^99m^Tc-methylene diphosphonate (Shanghai Syncor Pharmaceutical Co., Ltd., Shanghai, China) and static imaging was performed within 3–6 h. iii) HPO was diagnosed by experienced radiologists based on a diffuse, symmetric pattern of bilateral increased uptake in the long tubular bones on the bone scintigrams. Each image was independently examined by two specialists. iv) All the patients had digital clubbing in the extremities as well as joint pain.

### Statistical analysis

The χ^2^ test was applied to elucidate the differences between various groups. P<0.05 was considered to indicate a statistically significant difference. All statistical tests were analyzed using SPSS version 16.0 (SPSS, Inc., Chicago, IL, USA) and survival rates were calculated using the Kaplan-Meier method.

## Results

### Patient characteristics

Among the 6,151 patients with lung cancer who underwent bone scintigraphy, 183 exhibited an abnormally high uptake in the diaphysis and metaphysis of the tubular bones. Among the 183 patients, 115 had digital clubbing in the extremities and joint pain, which were confirmed as HPO.

**Figure 1:**
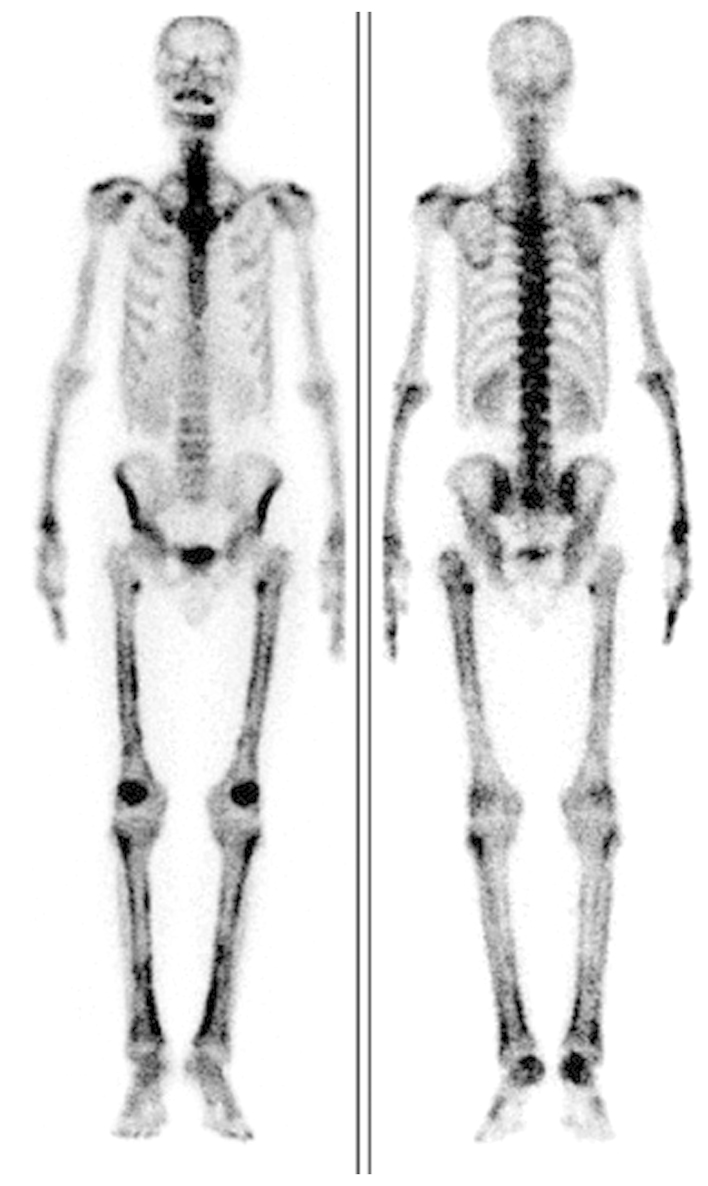
Bilateral femur uptake of ^99m^Tc-hydroxymethylene diphosphonate on bone scintigraphy.

Table I shows the clinical characteristics of the patients. There were 10 female and 105 male patients with a median age of 62 years (range, 31–78 years) and 87 patients had a history of smoking with 74 classified as heavy smokers (>30 pack-year). A total of 52 patients had a performance status of 0, 47 had a performance status of 1 and 16 exhibited a performance status of 2 or 3 (15). The histological diagnosis was adenocarcinoma in 55 patients, squamous cell carcinoma in 27, adenosquamous carcinoma in 11, large-cell carcinoma in three patients and two patients were diagnosed with small cell lung carcinoma. The other 17 patients were diagnosed with non-small cell lung cancer. The clinical stage was IIIB and IV in 31 and 33 patients, respectively, 10 were stage IB, five were stage IIA, five were stage IIB and 23 patients were stage IIIA. Two different patterns were observed on bone scintigraphy (Fig. 1); diffuse uptake, predominantly in the long bones of 72 patients (62.6%) and the other was uptake, predominantly in the joints of 43 patients (37.4%). Of the stage IIIB and IV patients, diffuse uptake accounted for 76.6% (49/64) and by contrast, only 52.9% (27/51) of the resectable patients (stage IA to IIIA) exhibited this pattern (P=0.029).

### Patient treatment and symptom improvement

A total of 56 patients underwent surgery, including 48 cases of radical excision and eight cases of palliative resection. No cancer-related mortalities were observed among the surgically resected group. Twenty-one patients underwent surgery alone, whilst 88 patients were treated with postoperative radiotherapy, chemotherapy or palliative treatment; six patients discontinued treatment.

The symptoms of HPO improved in 92 patients post-surgery or following chemotherapy. In patients with HPO who presented with joint pain at the inital diagnosis, the symptom was found to improve in 51/56 patients who underwent surgical resection and 41/53 patients who did not undergo surgery (not including the six patients that discontinued treatment). The improvement rate varied between the resected and non-resected patients (P=0.049). Bone scintigraphy was repeated in 85 patients within 3–6 months and the HPO symptoms improved in 70 patients.

### Survival analysis

The median follow-up time of the resected patient group and the non-resected patient group was 76 and 13 months, respectively. The overall five-year survival time was 10.2% in all 115 patients. The one-year overall survival time of the non-resected patients was 57% and their median survival time was 13.5 months. The median survival time was 35.7 months for the resected patient group.

## Discussion

Of the studies included in the present report, incidences of HPO ranged from 0.7 to 17% in lung cancer patients (Table II) (16). In the current study, HPO was presented with an incidence of 0.64%, which is lower compared with that of previous studies. To the best of our knowledge, this is one of the largest studies concerning HPO secondary to lung cancer.

The proportion of males is high and comprises 70–90% of the HPO population (17,18). A total of 105 (91.3%) patients with HPO in the present study were male. Histopathologically, adenocarcinoma predominated (47.8%), squamous cell carcinoma was second (23.5%) and there were only two cases of small cell carcinoma (1.7% of all lung cancer patients with HPO) in the present study. In previous studies, adenocarcinoma accounted for 11–53% of patients with lung cancer and HPO (Table II), whereas squamous cell carcinoma accounted for ~25–50%. The patients with clinical stage IIIB or IV (56%) disease predominated in the present study, and the patients treated with surgery accounted for 49% (Table I). Numerous studies have described that surgical resection frequently leads to immediate marked remission of joint symptoms and abnormal bone scintigram findings of HPO secondary to lung cancer (19,20). In the present study, the HPO symptoms promptly improved following surgical resection in 91.1% (51/56) of the patients with lung cancer. The improvement rate was 77.4% in non-resected patients, which was lower compared with the resected patients (P=0.049). The majority of symptoms were eliminated in patients following surgical resection, with a higher proportion than non-resected patients. Therefore, we suspect that the the existance of the tumor may influence HPO. This may be attributed to the affect of the lesion on HPO.

Despite numerous years of clinical diagnoses of HPO, the pathogenesis remains poorly understood. The predominantly investigated factor is biochemical; the biochemical hypothesis states that biochemical compounds are produced and released by the lung cancer itself. Elevated levels of serum growth hormone, growth hormone-releasing hormone, vascular endothelial growth factor and platelet-derived growth factor have been reported in certain patients with lung cancer and HPO (21–23). In 1987, Dickenson and Martin (24) hypothesized that pathological shunting around the pulmonary vasculature permits numerous circulating factors, which are normally inactivated by the lungs, to directly enter the systemic vasculature. This hypothesis may partly elucidate the proportional difference in symptom improvement between advanced and early-stage lung cancer patients. Uppal *et al* (25) mapped HPO to chromosome 4q33–q34 using autozygosity methods and revealed mutations in 15-hydroxyprostaglandin dehydrogenase, which is the primary enzyme of prostaglandin degradation. Homozygous individuals develop HPO secondary to chronically elevated prostaglandin E2 levels. These findings indicate that a mechanism operates at the gene level. Additional debatable hypotheses include mechanical and neurogenic reasoning (26). As the incidence of HPO is rare, future investigations are required to detect the true mechanism.

The standard treatment for HPO is resection of the primary lesion. In the case of advanced lung cancer, however, surgical excision of the tumor can be complicated if the underlying primary disease is not controlled by chemotherapy or radiation therapy. Administration of adrenocortical hormone is known to be a useful adjuvant treatment. Furthermore, radiofrequency ablation and epidermal growth factor receptor-tyrosine kinase inhibitor treatment have been reported and achieved favorable results (27,28).

As a retrospective observation, the present study has certain limitations. The patients that were included are cases who exhibited apparent clinical symptoms. Therefore, the sample size of HPO in the study may be less than the true incidence of such lesions. In addition, the mechanisms that predispose lung cancer to give rise to HPO were not detected.

In conclusion, this retrospective study of lung cancer patients that were treated at one cancer center, indicated that 1.87% of the 6,151 patients had symptoms of HPO. Males, adenocarcinoma patients, smokers and those with advanced disease contributed to the majority of cases of HPO in primary lung cancer patients. The symptoms and bone scintigram of HPO improved in the patients who received treatment, particularly in the patients that underwent surgery. Despite recent advances in recognizing and treating HPO, further investigations are required to elucidate its pathogenesis.

## Figures and Tables

**Table I:** Characteristics of 115 patients with lung cancer and hypertrophic pulmonary osteoarthropathy.

Characteristic	N (%)
Age, years	
Median	62
Range	31–78
Gender	
Male	105 (91)
Female	10 (9)
Smoker	
Current or ex-	87 (76)
Non-	28 (24)
Histology	
Adenocarcinoma	55 (48)
Squamous cell carcinoma	27 (23)
Adenosquamous carcinoma	11 (9)
Large cell carcinoma	3 (3)
Small cell carcinoma	2 (2)
Other	17 (15)
Stage	
IA	8 (7)
IB	10 (9)
IIA	5 (4)
IIB	5 (4)
IIIA	23 (20)
IIIB	31 (27)
IV	33 (29)
Treatment	
Yes	111 (97)
No	4 (3)
Surgery	
Yes	56 (49)
No	59 (51)
Symptoms	
Yes	39 (34)
No	76 (66)

**Table II:** Review of the characteristics of patients with lung cancer and hypertrophic pulmonary osteoarthropathy from previous studies and the present study (with sample sizes of >10 cases).

			Smoking index, pack-year		Histology		Clinical stage	
					
Author (Ref.)	Incidence^a^ (%)	Male/Female	<30 (%)	>30 (%)	Ad (%)	Non-Ad	IA–IIIA (%)	IIIB–IV (%)
Ray and Fisher (5)	13/149 (8.7)	-	-	-	3 (23)	10 (77)	-	-
Segal and Mackenzie (6)	16/1920 (0.8)	11/5	6 (38)	10 (63)	6 (38)	10 (63)	-	-
Morgan *et al* (15)	28/164 (17)	22/6	-	-	3 (11)	25 (89)	-	-
Ito *et al* (16)	19/2625 (0.7)	17/2	1 (5)	17 (89)	10 (53)	9 (47)	6 (32)	13 (68)
Izumi *et al* (17)	55/1226 (4.5)	39/16	-	-	25 (45)	30 (55)	-	-
Current study	115/6151 (1.9)	105/10	41 (36)	74 (64)	55 (48)	60 (52)	51 (44)	64 (56)

a Hypertrophic pulmonary osteoarthropy/total number of lung cancer patients;

Ad, adenocarcinoma.
